# Evaluation of the TRANSFORM Pilot Training Program for Community Health Workers and Traditional and Faith-Based Healers in Bangladesh

**DOI:** 10.1192/bjo.2024.231

**Published:** 2024-08-01

**Authors:** Tanjir Rashid Soron, Kazi Shammin Azmery, Farzana Akter, Belaluzzaman Rana, Maksudur Rahman

**Affiliations:** 1 Telepsychiatry Research and Innovation Network Ltd, Dhaka, Bangladesh; 2 NiHealth Ltd, Dhaka, Bangladesh

## Abstract

**Aims:**

In the densely populated Korail slum of Bangladesh, there is a critical gap in mental health care provision and utilization that was revealed in our ethnographic study. We observed the pivotal role of Community Health Workers (CHWs), Medicine Sellers, and Traditional and Faith-Based Healers (TFHs) in the existing health care service delivery. Moreover, we explored the opportunity to collaborate with them to ensure universal access to biomedical care for serious mental disorders in this slum. As a part of this collaborative approach, we aimed to train these 4 key stakeholders through co-designed training programs that were codeveloped through extensive community engagement including 5 co-designing workshops and 2 writing workshops with them. Furthermore, we refined the initial training program by an expert committee and stakeholders. This training program was piloted to find out the acceptability, feasibility, impact, challenges and areas of improvement.

**Methods:**

We followed mixed-methods approach to evaluate the 3-day pilot training with 20 participants at Mirpur, Dhaka. In quantitative part of evaluation we used a) pre and post test assessment that has been carefully designed to assess knowledge, skills, communication, attitudes and motivation, b) session specific questionnaire to find out feedback of the content, activities and time sensitivity of the session, anonymous feedback forms.

In the qualitative part, we conducted a) focus group discussions (FGDs) after completion of training with each group, b) observational notes from each session for deeper understanding.

**Results:**

The pilot training engaged a diverse group of 20 participants and their age ranged from 24 to 52 years, representing 11 different organizations. Though most of the participants were working in the health sector for a long time, we found more than 10% of the participants believed there was no effective biomedical care for the serious mental disorder during pretesting. However, their perception changed during the training. The role playing and case scenario was the most engaging and enjoyable part. We found the participants considered their knowledge regarding the mental health increased up to 80% from their baseline. Our research team also found the increased number of referrals to the biomedical care from the community after the pilot training.

**Conclusion:**

The increased motivation and sense of responsibility reported by participants underscore the training program's effectiveness and the experience and learning from this pilot helped us to further refinements of the training program for the traditional and faith based healer, community health workers and medicine to transform the mental health scenario in Bangladesh.

